# Preparation and Structural-Thermodynamical Investigation of Renewable Copolyesters Based on Poly (Ethylene Succinate) and Polyisosorbide

**DOI:** 10.3390/polym16152173

**Published:** 2024-07-30

**Authors:** Chaima Bouyahya, Panagiotis A. Klonos, Alexandra Zamboulis, Eleftheria Xanthopoulou, Nina Maria Ainali, Mustapha Majdoub, Apostolos Kyritsis, Dimitrios N. Bikiaris

**Affiliations:** 1 Laboratory of Polymer Chemistry and Technology, Department of Chemistry, Aristotle University of Thessaloniki, 541 24 Thessaloniki, Greece; bouyahyachaima@fsm.u-monastir.tn (C.B.); azampouli@chem.auth.gr (A.Z.); nsainali@chem.auth.gr (N.M.A.); 2 Laboratoire des Interfaces et Matériaux Avancés, Université de Monastir, Monastir 5000, Tunisia; mustapha.majdoub@fsm.rnu.tn; 3 Department of Physics, National Technical University of Athens, Zografou Campus, 157 80 Athens, Greece; akyrits@central.ntua.gr

**Keywords:** poly(ethylene succinate), poly(isosorbide succinate), renewable copolyesters, structure-properties relationships, molecular dynamics

## Abstract

A series of novel renewable copolymers based on poly(ethylene succinate) (PESu) and poly(isosorbide succinate) (PISSu), with the Isosorbide (Is)/PESu molar ratio varying from 5/95 to 75/25, were synthesized in-situ and studied in this work. A sum of characterization techniques was employed here for the structural and thermo-dynamical characterization. The sophisticated technique of dielectric spectroscopy, along with proper analysis, enabled the molecular dynamics mapping of both the local and segmental types, which is presented for such materials for the first time. With increasing the Is fraction, shorter copolymeric entities were gradually formed. Based on the overall findings, the systems were found to be homogeneous, e.g., exhibiting single glass transitions, with the two polymer segments being found to be excellently distributed. The latter is indirect, although strong, evidence for the successful copolymerization. The thermal degradation mechanism for the copolymers was exhaustingly explored employing analytical pyrolysis. The systems exhibited, in general, good thermal stability, according to the thermogravimetric analysis. Confirming one of the initial scopes for the present systems, isosorbide plays here the role of hardener (PISSu) over the soft polymer (PESu), and this is reflected in the monotonic increase of the glass transition temperature, *T*_g_, from −16 to ~56 °C. The introduction of Is results in an increase in constraints (hardening of the matrix), while there seems to be an overall densification of the polymer (decrease of the free volume).

## 1. Introduction

For more than a century, polymers, commonly referred to as plastics, have been applied to a vast number of human needs, namely, from industry to biomedicine, academia, and everyday life [1,2]. This mainly owes to the fact that polymeric materials combine, in general, a sum of wanted properties regarding the requirements of applications with the low cost of synthesis and processing [3,4]. As expected, due to the growing aspects/needs of humanity for new and more targeted applications (e.g., aeronautics, drug delivery, 3–4D printing, etc.), more and more specialized materials are necessary. In parallel, along the fast development of society, there have grown significant concerns for the large amount of ‘plastic waste’ on earth [5,6], which has been, up to now, due to the extensive use of polymers synthesized from fossil-based sources and employing non-ecofriendly processes [7]. A main problem for this accumulation of traditional polymer wastes is their lack or weak degradability in nature [8,9,10,11]. Obviously, this is combined with the lack of people’s general environmental consciousness [12]. During the last couple of decades, such consciousness has grown, and, regarding polymers, a serious attempt to turn toward the so-called ‘green materials’ and ‘circular economy’ is taking place [13,14,15].

A partial solution to these issues is attempted via the use of polymers based on renewable resources [16,17,18,19,20], for example, by exploiting agricultural products and byproducts. Such can be, for example, acids formed during metabolic processes (lactic acid, succinic acid, furan-dicarboxylic acid, adipic acid, caprolactone, vanillic acid, itaconic acid, etc.) [16,21,22,23,24,25]. Among others, the polymers prepared by such resources, being mainly polyesters [26,27], exhibit both desired properties, such as thermochemical stability, good mechanical and permeation performance, and good and moderate physical/enzymatic degradation rates in soil conditions (compostability) [28]. As a next step, there has been progress by many groups, including ours, to combine such biobased polymers in the form of polymeric blends [29,30,31] and copolymers [32,33,34,35,36,37], as well as composites/nanocomposites [38,39,40,41]. This combination has opened room for new innovative materials envisaged for even more specific applications due to the achievement of complex and, even, new properties of polymers. For example, this work has resulted in the almost ‘at will’ manipulation of thermal degradation, mechanical performance, crystallinity, permeation of small molecules [42], thermal and electrical conductivity [43], as well as physical degradation [44]. In the same context, the study of these new materials has been fruitful from the point of view of basic research.

In our recent published works, we have shown that by carefully chosen synthetic routes, the compatibilization of different polyesters can be achieved by in situ polymerization of a polymer block in the presence of either another existing block or simultaneously with the polymerization of the second block [34,36,45,46]. The compatibilization and successful copolyester synthesis result in many effects, for example, the tuning of the glass transition, crystallization and melting, the manipulation of free volume [47], and, subsequently, the micro- and macro-scopic performance of the polymers.

Following such routes, in the present study, we synthesized and investigated a series of copolymers based on two aliphatic renewable polyesters. On the one hand, poly(n-alkylene succinate) [48,49,50] with *n* = 2, i.e., poly(ethylene succinate) (PESu), is used as the first block. On the other hand, poly(isosorbide succinate) (PISSu) [51,52] was synthesized as the second block.

We should report that isosorbide is a diol exhibiting a ‘V’-like shape consisting of two sterically different secondary hydroxyl groups with the so-called ‘exo-’ and ‘endo-’ configurations, respectively, as well as two ‘cis-’ connected tetrahydrofuran rings [53,54,55]. Isosorbide is actually a commercial biobased monomer, produced either from D-glucose or, even, from starch [56]. Isosorbide has been involved in part in the step-type synthesis of many polymers, including polyesters, polyurethanes, polyamides, polycarbonates, etc. [56,57,58,59].

Coming back to the present systems, for PISSu, prior to polymerization, the isosorbide (Is) monomers were practically mixed with the succinic acid at various Is/ethylene glycol molar ratios between 5/95 and 75/25. For comparison, we also synthesized the two homopolymers PESu and PISSu, i.e., of molar ratios 0/100 and 100/0, respectively. For the structural investigation of these newly synthesized co-polyesters, we employed infrared spectroscopy, nuclear magnetic resonance spectroscopy, gel permeation chromatography, thermogravimetric analysis, and pyrolysis gas chromatography-mass spectrometry analysis, while for the thermal transitions, we employed conventional calorimetry. Finally, to investigate molecular mobility (local and segmental), we employed the sophisticated technique of broadband dielectric spectroscopy. Upon analysis of the results and evaluation by widely adopted routes and models, we combined the results by the different techniques to conclude the in-depth structure-properties relationship. Especially, the molecular dynamics for these novel systems are shown here for the first time.

## 2. Materials and Methods

### 2.1. Materials

Succinic acid (SA, purum, ≥99.5%), ethylene glycol (EG, 99.8%), and isosorbide (Is, 98%) were purchased from Sigma Aldrich (St. Louis, MO, USA). Titanium isopropoxide (TIS, ≥97%), used as a catalyst, was provided by Alfa Aesar (Kandel, Germany). Any other employed reagents were of analytical grade.

### 2.2. Synthesis of Copolyesters

**Figure 1:**
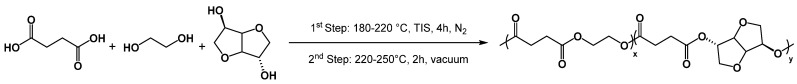
Schematic of the synthesis of poly(ethylene succinate)-co-(isosorbide succinate).

The PESu-PISSu (Figure 1) copolyesters were prepared by a two-step in-situ melt polycondensation method (esterification and polycondensation). During the esterification process, appropriate amounts of SA, EG, and Is with 400 ppm of titanium isopropoxide (TIS) catalyst were added to a 250 mL round-bottom flask equipped with a mechanical stirrer and a vacuum apparatus. The reaction mixtures were degassed and purged three times with dry nitrogen. The reaction mediums were then heated up to 180 °C for a period of time until all the reagents were completely melted. Then, the reaction mixtures were further heated at a slow rate to 220 °C and maintained there for 4 h. During the polycondensation process, a high vacuum was applied, and the temperature was increased slowly to 250 °C for 2 h.

In total, eight (8) polyesters were prepared, namely, six (6) copolyesters with Is/EG mol ratios of 4.5/95.5, 9.6/90.4, 18.6/81.4, 35/65, 50/50, and 75/25, and two (2) homopolymers, namely, neat PESu (0/100) and neat PISSu (100/0). For the sake of ease, a specific code name is used henceforth. The list of samples, along with the code names and main properties, can be found in Table 1 and Table 2 (sections in the following).

### 2.3. Fourier-Transform Infrared Spectroscopy (FTIR)

For the FTIR study we employed a Perkin-Elmer spectrometer (Thermo Fisher Scientific, Waltham, MA, USA). Each sample was scanned 32 times with a resolution of 4 cm^−1^ from 4000 to 650 cm^−1^. All spectra were subsequently baseline-corrected and normalized.

### 2.4. Nuclear Magnetic Resonance Spectroscopy (NMR)

Nuclear magnetic resonance spectra were recorded at room temperature, in deuterated chloroform, on an Agilent 500 spectrometer (Agilent Technologies, Santa Clara, CA, USA). Spectra were calibrated using the residual solvent peaks. The average sequence length and degree of randomness were calculated according to Equations (1) and (2).

(1) $$L_{EGSu}=1+\frac{2\times I_{EGSuEG}}{I_{EGSuIs-EG}+I_{EGSuIs-Is}}\;\mathrm{and}\;L_{IsSu}=1+\frac{2\times I_{IsSuIs}}{I_{EGSuIs-EG}+I_{EGSuIs-Is}}$$

(2) $$R=\frac{1}{L_{EGSu}}+\frac{1}{L_{IsSU}}$$

where *L_EGSu_* and *L_IsSu_* are the average sequence lengths of the copolymer segments containing EG and Is respectively, *I_EGSuEG_* is the integration of the C(O) resonance signals of succinic acid with EG units on both sides, *I_IsSuIs_* is the integration of the C(O) resonance signals of succinic acid with Is units on both sides, *I_EGSuIs-EG_* is the integration of the C(O) resonance signals of the carbon atom adjacent to EG in units of succinic acid with EG and Is on each side, *I_EGSuIs-Is_* is the integration of the C(O) resonance signals of the carbon atom adjacent to Is in units of succinic acid with EG and Is on each side, and *R* is the degree of randomness.

### 2.5. Gel Permeation Chromatography (GPC)/Size Exclusion Chromatography (SEC)

The number and weight (average molecular weight, respectively, Mn and Mw) and dispersity (PDI) of synthesized materials were obtained by GPC/SEC. To that end, we employed an Agilent 1260 Infinity II LC system (Agilent Technologies, Santa Clara, CA, USA) equipped with an isocratic G7110B pump, an automatic vialsampler G7129A, a Refractive Index Detector (RID) G7162A, and a PLgel 5 μm (50 × 7.5 mm) guard column combined with 2 PLgel 5 μm (300 × 7.5 mm) MIXED-C columns. Polystyrene standards with Mns varying between ~0.5 and ~1600 kg/mol were used for the determination of the calibration curve. The corresponding solutions were prepared at a concentration of 3 mg/mL and filtered through PTFE filters of ~0.45 μm in pore size. The injection volume was 20 μL, whereas the total elution time of each sample was 30 min. The temperature of the columns and the RID were both fixed at 40 °C.

### 2.6. Differential Scanning Calorimetry (DSC)

The thermal transitions, with a focus on the glass transition, were studied by DSC in the temperature range of −100 to 160 °C and in a high-purity nitrogen atmosphere. The measurements were conducted by means of a TA Q200 calorimeter (TA Instruments, New Castle, DE, USA) properly calibrated for temperature and heat capacity. Pieces of the prepared samples of ~8 mg in mass were closed in TA-standard aluminum pans. All samples were subjected to a first heating at 160 °C to erase any thermal history and maximize thermal contact with the aluminum pan. Then, a cooling and subsequent heating scan were performed at the fixed rate of 10 K/min.

### 2.7. Broadband Dielectric Spectroscopy (BDS)

The technique of dielectric spectroscopy was employed to assess the local and segmental molecular dynamics. To that end, we employed a Novocontrol (GmbH, Germany) BDS setup, i.e., an Alpha frequency response analyzer combined with a Quatro liquid-nitrogen cryosystem. The measurements were performed on samples in the form of a sandwich-like capacitor, with the polymer sample (insulator) being melted between two well-polished, brash disk-like plates (electrodes, one with a diameter of 14 mm and the other of 30 mm). The specimen diameter was determined by the upper electrode diameter (14 mm). To prevent the contact of the electrodes and keep a constant electrode’s distance, thin silica spacers from Novocontrol (~100 μm) were employed. The melting of the polymers was performed on a hot plate by heating the samples up to *T* equal to the melting temperature (*T*_m_), the latter being previously estimated by DSC. The samples were kept at *T*_m_ for ~30 s, removed from the hot plate, and immediately placed on a large, cold metallic surface in order to be cooled down as soon as possible. After this process, the capacitor was placed in a Novocontrol BDS 1200 measurement cell and inserted into the cryostat. Prior to the recordings, the sample was cooled in the cryostat by a cold nitrogen gas flow down to −140 °C. The complex dielectric permittivity, ε*, was isothermally recorded as a function of frequency, from 0.1 Hz to 1 MHz, for a large range of temperatures, from −140 to 120 °C, during heating at steps of 5 or 10 K, depending on the followed process.

### 2.8. Thermogravimetric Analysis (TGA)

The thermal degradation profile of the synthesized copolymers was evaluated by thermogravimetric analysis (TGA), employing the thermal analyzer NETZSCH STA 449F5 (Germany). At a heating rate of 20 °C/min, the samples were heated in the temperature range of 30 to 600 °C under N_2_ atmosphere (99.9%) at 30 cm 3 min^−1^.

### 2.9. Pyrolysis–Gas Chromatography-Mass Spectrometry Analysis (Py–GC/MS)

For Py-GC/MS analysis [60] of the PESu-PISSu copolymers, a small amount of each composition/sample was initially placed into the “Double-Shot” EGA/PY 3030D Pyrolyzer (Frontier Laboratories Ltd., Fukushima, Japan) employing a CGS-1050Ex carrier gas selector. For the pyrolysis analysis (flash pyrolysis), each sample was placed into the sample cup and, subsequently, allowed to fall into the Pyrolyzer furnace. The temperature of the preselected pyrolysis was set at 450 °C, while the GC oven temperature was programmed at 50 °C for 2 min. This was followed by a step-like elevation of the temperature to 200 °C at 5 °C/min, at which the temperature was kept stable for a period of 8 min. Then, the temperature was increased to 300 °C at 20 °C/min, where it was preserved for 5 min. Sample vapors generated in the furnace were split (at a ratio of 1/50), a portion moved to the column at a flow rate of 1 mL/min and a pressure of 53.6 kPa, and the remaining portion exited from the system via the vent. The pyrolyzates were separated using a temperature-programmed capillary column of a Shimadzu QP-2010 Ultra Plus (Kyoto, Japan) gas chromatogram and analyzed by the mass spectrometer MS-QP2010SE of Shimadzu (Kyoto, Japan). An Ultra-ALLOY^®^ metal capillary column from Frontier Laboratories LTD (Fukushima, Japan) was used, containing 5% diphenyl and 95% dimethylpolysiloxane stationary phases, a column length of 30 m, and a column ID of 0.25 mm. For the mass spectrometer, the following conditions were used: ion source heater 200 °C, interface temperature 300 °C, vacuum 10^−4^–10^0^ Pa, *m*/*z* range 40–500 amu (atomic mass unit), and scan speed 10,000. The ion gas chromatograms and spectra retrieved by each experiment were subjected to further interpretation through Shimadzu and Frontier post-run software. The chromatograms and spectra retrieved by each experiment were subjected to further interpretation through Shimadzu (NIST11.0) and Frontier (F-Search software 4.3) post-run software. The identification/recognition were done depending on the similarity percentage (minimum value of 80%) between the average mass spectra on the entire chromatogram.

## 3. Results and Discussion

### 3.1. Materials—Scope—Structure

The initial scope of these materials (described in Table 1) is the tuning of PESu properties (soft polymer, low glass transition temperature polymer) by the addition of another harder monomer: isosorbide. The copolymerization involving the in-situ polymerization of the different monomers simultaneously aims at achieving the as-uniform as possible distribution (compatibilization) of the two polymers.

**Figure 2:**
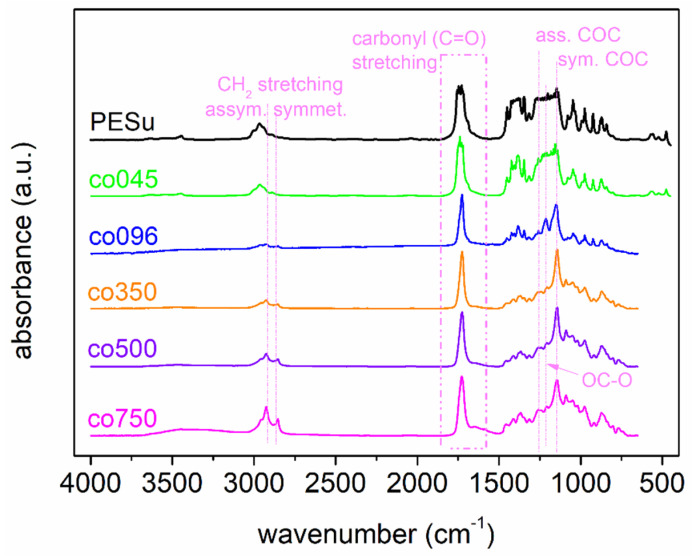
Comparative FTIR spectra for PESu and selected PESu/PISSu copolymers. The most significant absorbance bands along the corresponding molecular-bond origins are marked within the plot.

The structural characterization begins with FTIR, the results of which are shown in Figure 2. The main absorbance peaks are described within the plot. These are the CH_2_ symmetric and asymmetric stretching, the various O-C-O and C-O-C vibrations, and the vibration of the ester bond (-C=O), the latter being the more polar site of PESu and is expected to be involved within interpolymer physical associations/interactions. Regarding PESu, the FTIR data are in accordance with previous findings in the literature [61,62].

**Figure 3:**
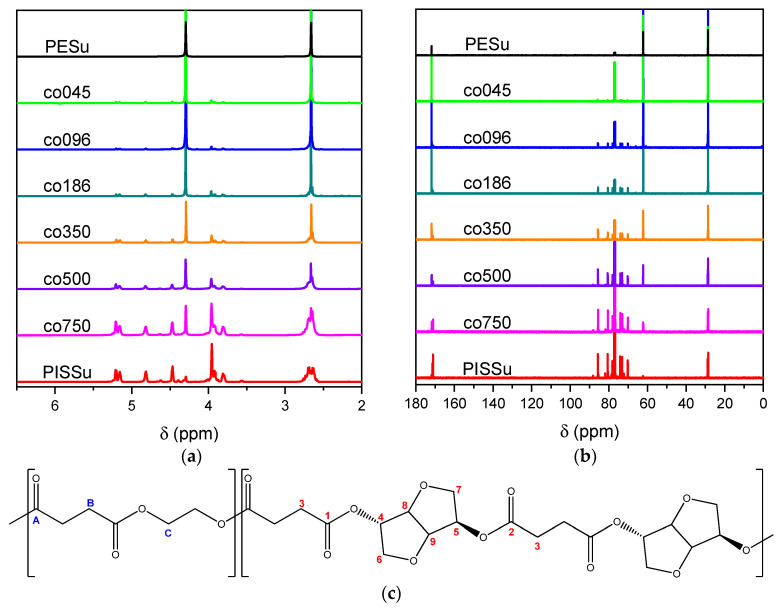
Nuclear magnetic resonance spectra of the synthesized polymers, (**a**) ^1^H and (**b**) ^13^C, and (**c**) the structure of the copolymers.

The NMR spectra of the homo- and copolymers are presented in Figure 3. In the 1H NMR spectrum of PESu, two resonance signals corresponding to OCH_2_ C (4.29 ppm) and C(O)CH_2_ B (2.66 ppm) groups are observed. In the spectra of PISSu, we can note the following signals: 5.20 and 5.16 ppm (OCH 4&5), 4.83 and 4.47 ppm (CH 8&9), 3.9 and 3.8 ppm (CH_2_ 6&7), 2.7 ppm (C(O)CH_2_ 3). Signals of both PESu and PISSu segments are observed in the copolymers, with proportional intensities to the EG/IS ratio, thus confirming the successful polymerization. Similarly, in the 13C spectra of the copolymers, signals of both PESu and PISSu segments are observed: 171.8 ppm (C(O) A), 171.4 and 171.2 ppm (C(O) 1&2), 85.7 and 80.6 ppm (CH 8&9), 78.1 and 74.1 ppm (OCH 4&5), 73.1 and 70.3 ppm (CH_2_ 6&7), 62.2 ppm (OCH_2_ C), and 28.6–28.8 ppm (C(O)CH_2_ 3&B).

Regarding the composition of the copolymers (calculated by comparing the integration of the -OCH- signals of the PISSu segments and the -OCH_2_- signals of the PESu segments), we can note that the Is content is slightly lower compared to the feed ratio, and this trend is more pronounced in the copolymers with a higher Is content. Finally, the microstructure of the copolymers was studied by NMR (Table 1), and co350, co500, and co750 were found to be random copolymers (*R* = 1). For the other copolymers synthesized with a lower Is content, i.e., co045, co096, and co0186, the studied NMR signals could not be safely integrated; the copolymers are probably constituted by large PESu blocks interrupted by random PISSu units. More specifically, the ester carbon atoms exhibit slightly different shifts depending on whether the adjacent units are an EG or an Is unit. Therefore, besides the EG-SA-EG and Is-SA-Is units, where succinic acid is esterified by the same alcohol, EG or Is, discrete signals corresponding to EG-SA-Is or Is-SA-EG units were detected. Assuming similar relaxation times for all C=O ester groups, the average sequence length, *L*, and degree of randomness of the copolymers were calculated [63].

**Figure 4:**
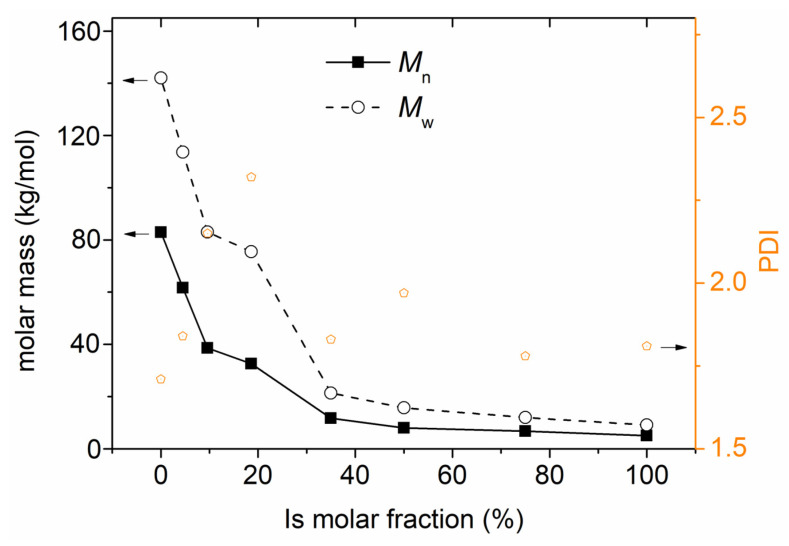
The isosorbide molar fraction dependence of (left axis) the molar mass values *M*_n_ and *M*_w_ and (right axis) of the polydispersity index.

In Figure 4, we present the results by GPC-SEC for *M*_n_/*M*_w_ and PDI. Neat PESu exhibits an *M*_n_ of ~83 kg/mol and a moderate *PDI*~1.7. During the copolymerization, the *M*_n_ drops systematically, with the Is fraction increasing. This can be explained as follows: Is hydroxyl groups have a lower reactivity compared to EG. Since the time periods and temperatures of the polymerization were kept constant for all systems, we expected the formation of gradually shorter copolyesters when increasing Is due to its lower reactivity, as indicated by the systematic *M*_n_ drop. We have reported similar correlations of *M*_n_ alterations with successful/homogeneous in situ copolymerization within various other copolyesters (e.g., in [34,35,36,46]). At the same time, within all copolyesters, *PDI* is increased, interestingly, in a non-monotonic way regarding the Is ratio. Obviously, the situation of the chemo- and thermodynamical processes taking place is quite complex to easily interpret the complex *PDI*(Is) trend.

### 3.2. Glass Transition—Thermal Events

**Figure 5:**
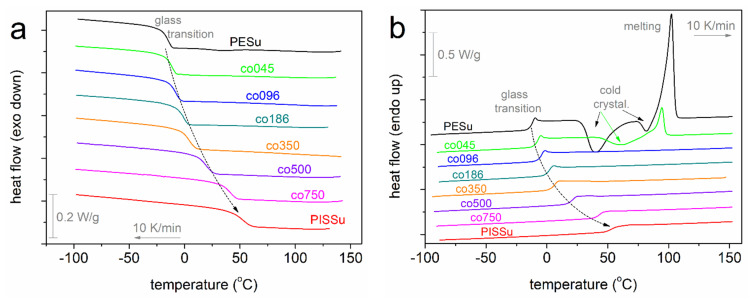
Raw DSC results for all samples are indicated during (**a**) cooling and (**b**) subsequent heating. The recorded heat flow is shown upon normalization to each sample mass. The added curved arrows mark the effect on the glass transition step by increasing the isosorbide molar fraction.

Coming to the thermal response, we present in Figure 5 the comparative DSC traces for all compositions. We recall that prior to the cooling shown in Figure 5a, all samples had been well melted at ~160 °C; thus, any thermal history was erased and the thermal contact between the polymer and the aluminum pan was optimized. Neat PESu exhibits a weak crystallization peak at ~34 °C (Table 2), whereas PISSu is amorphous. The presence of Is in the copolymers, at all ratios, seems to preclude the weak crystallization of PESu, as manifested by the complete lack of exothermal peaks in the copolymers.

During cooling as well as the subsequent heating (Figure 5b), all samples demonstrate single glass transition steps. This provides, by itself, solid evidence for the homogeneity of the systems. From the data on heating, we evaluated the characteristic temperature of the glass transition, *T*_g_, as the *T* of the half-heat capacity change, Δ*c*_p_/2. The *T*_g_ values are listed in Table 2 and are shown in Figure 5a as a function of the Is molar fraction. While for neat PESu, *T*_g_ equals −16 °C, in the copolymers, *T*_g_ increases monotonically up to 40 °C, tending to approach the value of neat PISSu (51 °C). To check this systematic behavior, we fitted mathematical models describing the degree of miscibility judging from the *T*_g_. Our results seem to coincide quite well with the prediction by the well-known Fox equation [64] for completely miscible polymers in the form of blends. A similar systematic dependence is observed for the Δ*c*_p_ of the glass transition (the so-called ‘glass transition strength’), dropping from 0.65 J/g∙K down to 0.46 J/g∙K.

**Figure 6:**
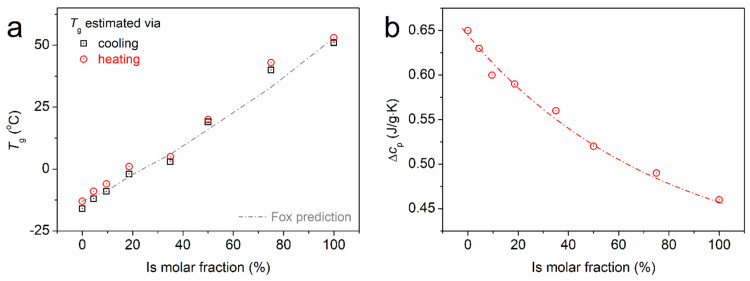
The isosorbide molar fraction dependence of (**a**) *T*_g_ and (**b**) Δ*c*_p_. The added line in (**a**) corresponds to the prediction by the Flory equation, whereas the added line in (**b**) is just an eye-guiding line.

From the point of view of segmental polymer mobility, both results (*T*_g_ and Δ*c*_p_, Figure 6) indicate that these PISSu/PESu can be considered homogeneous systems of excellently mixed polymers (miscible), wherein the ‘hard’ polymer (isosorbide-based) systematically affects the softer one (PESu). In practice, we gain the first significant proofs of the successfully synthesized copolymers and the achievement of the initial scopes. The simultaneous increase of *T*_g_ and decrease of Δ*c*_p_ is a result of constrained polymer chain mobility [65,66] (even the degrees of freedom for each chain), which actually suggests the hardening of the polymeric matrix. Obviously, this will have a severe impact on the materials’ performance, such as the mechanical permeation of small molecules (e.g., air, humidity, gases), heat transport, etc., as well as future processing.

Due to the hardening of the systems in combination with the increased rigidity of the copolymer chains, both due to the existence of the hard PISSu segments and the simultaneous shortening of the chain lengths, we would expect a suppression of crystallizability. This is found to be true, as observed by the almost immediate vanishing of melt/cold crystallization and melting events in Figure 5a,b. The result is another supportive clue on the copolymers’ homogeneity, as, at least on the micrometric scale, there seems to be no PESu separation from PISSu.

### 3.3. Molecular Dynamics

The effects on molecular dynamics of local and segmental types are assessed by BDS. Upon keeping fixed thermal treatment and initial erasing of the thermal history for all samples, we basically exploit the information supplied by the imaginary part of dielectric permittivity *ε*″(*f*,*T*). *ε*″ is considered to be related to the dielectric losses, whereas the dipolar relaxation mechanisms related to the molecular mobility are recorded as peaks of the isothermally recorded *ε*″(*f*) spectra.

**Figure 7:**
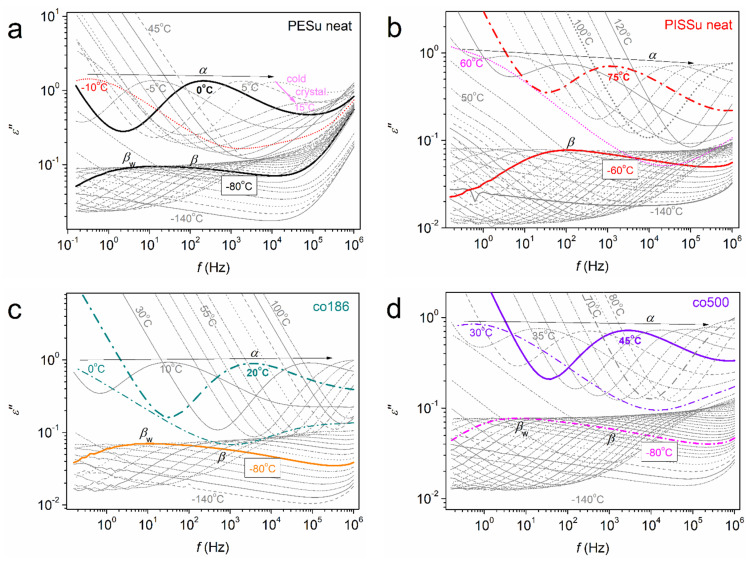
The raw BDS data for the indicated selected samples, namely, in terms of the isothermal *ε*″(*f*) plots at all temperatures of measurement. The results are shown for (**a**) neat PESu, (**b**) neat PISSu, (**c**) co186 and (**d**) co500. The local (*β*, *β*_w_) and segmental (*α*) relaxations, recorded as peaks of *ε*″, are indicated at selected temperatures.

In Figure 7, we present a selection of representative BDS data for the overall temperature range of investigation (−140 to 120 °C). Therein, mainly three distinct types of relaxations are recorded. At *T* < *T*_g_, the dipolar response is mainly weak and is dominated by two local-like relaxations, i.e., the ones named as *β* and *β*_w_. These relaxation mechanisms originate from the more localized molecular motions [35,67,68,69] (and references therein). At *T* > *T*_g_, the dielectric signal increases significantly by ~1 order of magnitude due to the implementation of additional dipole moments due to the mobilization of the overall polymer chains and their cooperative motions (transition from the glassy to rubbery state of the polymers). The corresponding relaxation mechanism (peak) is widely addressed as the ‘main *α* relaxation’ and is considered the dielectric and dynamic analogue of the calorimetric glass transition [70].

**Figure 8:**
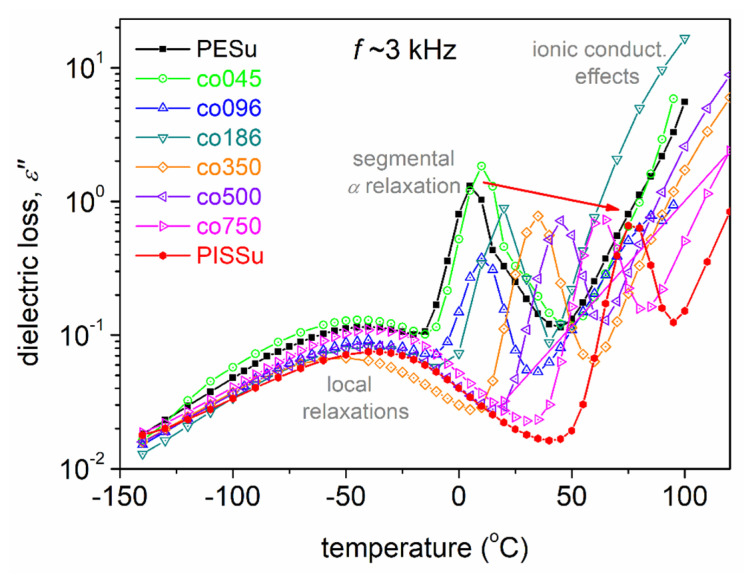
Comparative isochronal *ε*″(*Τ*) results for all compositions shown at 3.162 kHz. The added red arrow marks the impact of the isosorbide molar fraction on the main *α* relaxation.

To facilitate a better comparison between the different samples and, simultaneously, with the calorimetric findings (Figure 5b), we replotted the isothermal results [*ε*″(*f*)] in the form of isochronal plots [*ε*″(*T*)]. In particular, this is done for the selected relatively high frequency of ~3 kHz. These spectra can be seen in Figure 8. From these data, it can be easily seen that while the local dynamics (*β*-type relaxations) are barely affected by the copolymers’ composition, the segmental dynamics (α relaxation) migrate systematically toward higher temperatures with an increasing Is/EG ratio. This is in qualitative agreement with the effects recorded on the calorimetric glass transition, as shown in Figure 5b and Figure 6a. This migration suggests the retardation, or else deceleration, of segmental dynamics.

In order to enrich our conclusions with more information regarding molecular dynamics, such as the dynamical *T*_g_ and fragility, the width of the relaxation time range, and dielectric strength, we performed a specific analysis of the complex spectra in terms of fitting mathematical models [70,71]. To this end, we employed the Havriliak–Negami (HN) [72] function, mathematically described in Equation (3).

(3) $$\varepsilon^{*}\left(f\right)=\frac{\Delta \varepsilon }{{\left[1+{\left(\frac{if}{f_{0}}\right)}^{\alpha_{HN}}\right]}^{\beta_{HN}}}+\varepsilon_{\infty }$$

In the HN function, the following parameters are involved: the dielectric strength, Δε, a characteristic frequency related to the frequency of maximum dielectric loss, *f*_0_, a value of the real part of the dielectric permittivity (*ε*′) at *f* >> *f*_0_, *ε*_∞_, the shape parameter that denotes the width of relaxation times range, *α*HN, and, finally, another shape parameter that evaluates the symmetry or asymmetry of the *ε*″(*f*) peak, *β*_HN_. For *β*_HN_ < 1, the HN term is asymmetric, which is the common case for an amorphous, unaffected segmental *α* relaxation. We note that in the case of a symmetric relaxation, *β*_HN_ equals 1, and the HN equation becomes practically the so-called Cole-Cole function [73]. During the fitting, there is also the need to simulate the effects arising from conductivity-related effects, i.e., at the higher frequency side of the measurement window. For that, we may employ the additional linear term $-i\cdot \sigma_{0}/{\left(2\pi f\right)}^{C}\varepsilon_{0}$ where *σ*_0_ is related to the specific DC conductivity and the *C* exponent parameter describes the Ohmic (for *C* = 1) and non-Ohmic contributions in conductivity (for *C* < 1). In case of additional polarization effects, such as the ‘electrodes polarization’ additional terms may be needed [71,74].

**Figure 9:**
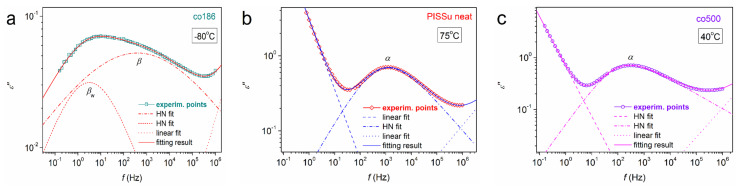
Examples of analysis of the *ε*″(*f*) spectra for three cases of samples [(**a**) co186, (**b**) PISSu neat and (**c**) co500] and temperatures in terms of model functions (details in the main text).

In Figure 9, we show representative examples of the performed fittings for both homopolymers and copolymers, as well as both local and segmental relaxations. As expected [70,71], the local-*β*-type relaxation could be sufficiently fitted by symmetric terms, whereas the segmental *α* relaxations were fitted by asymmetric HN terms.

**Figure 10:**
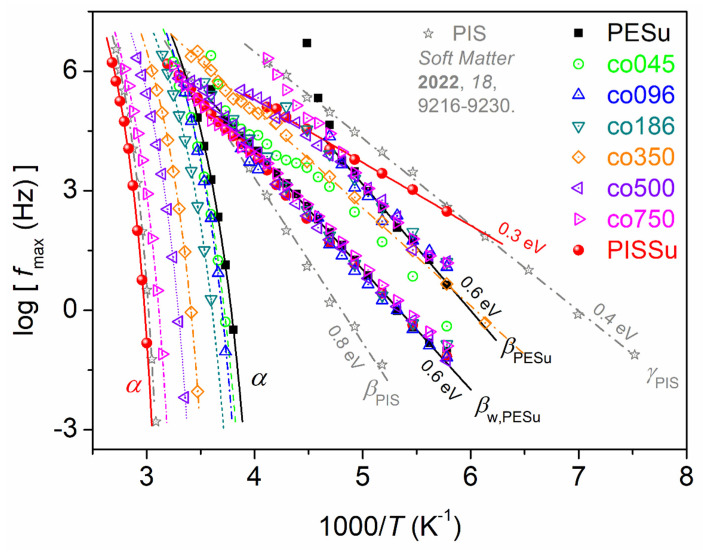
The overall dielectric relaxation map for all samples in terms of time scale, i.e., the reciprocal temperature dependence of the frequency maxima for all the resolved dipolar relaxations (local and segmental). For comparison, we have added results for neat poly (isosorbide) and PIS (grey stars), adapted from a recent work [36]. The straight and curved lines connecting the experimental points are fittings of the Arrhenius and the Vogel–Tammann–Fulcher–Hesse [75] equations, respectively.

From the values for the peak maxima, fmax, coming from this fitting, we were able to construct the dielectric relaxation map (time scale) by plotting log*f*_max_ as a function of the reciprocal temperature, 1000/*T*, for all samples under investigation. This map, also called Arrhenius plots, is presented in Figure 10. Therein, we have included data for neat poly(isosorbide) (PIS), as adapted from a recent work by our groups [36].

Comparing with previous findings on poly(*n*-alkylene succinates) [35,64,68], we could identify the local relaxations recorded here. Both processes were found to originate from the localized motions of PESu, namely the *β*_PESu_ and the *β*_w,PESu_. *β*_PESu_ has been proposed to monitor the dipole moments arising from the crankshaft rotations of the ester groups at the polymer backbone. Such relaxations in many polyesters have been found to depend strongly on segmental dynamics, in particular in poly(*n*-alkylene succinates) to even scale with the *T*_g_ [68], despite their local character. Regarding the slightly slower *β*_w,PESu_ relaxation, there are only two previous recordings in the literature on poly(*n*-alkylene succinates) to compare with [35,64,68]. The relaxation has been found to be stronger for small *n*- and weaker for larger *n*-alkylene sequencies. Based on similarities recorded between this process and other local processes arising from hydrophilic sites with attached water molecules located at solid surfaces and/or hydrophilic polymer groups [76,77,78], we have proposed such origins for our β_w,PESu_ [68].

For PESu and the copolymers, both processes exhibit independence from the Is fraction and show similar and almost constant activation energies, *E*_ac_t~0.6 eV. Interestingly, in neat PISSu, the local relaxation (*β*_PESu_-like process) is mainly accelerated, exhibiting a suppressed *E*_act_~0.3 eV. Taking into account the data for *γ*_PIS_ relaxation, it seems that *β* in PISSu could be the superposition of *β*_PESu_ and *γ*_PIS_. Contrariwise, *β*_w,PESu_ demonstrates a similar time scale with PESu and PESu/PISSu.

None of the local processes was found to coincide with the time scale of local relaxations of neat PIS (grey stars in Figure 10).

**Figure 11:**
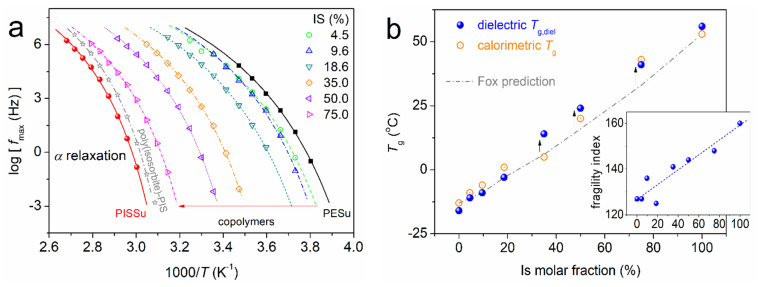
The segmental dynamics characteristics, in terms of (**a**) the time scale for *α* relaxation and (**b**) the isosorbide fraction dependence of the dielectric and calorimetric *T*_g_, as well as the fragility index of *α* relaxation, *m*_α_ (inset to **b**).

The focus is now turned to segmental dynamics. To facilitate the discussion, we created Figure 11a, which shows the dynamics map solely of the *α* relaxations. It is quite clear from a simple glance that by increasing the amount of the ‘hard phase’ (Is), the α relaxations are systematically decelerated, i.e., migrate toward higher temperatures/lower frequencies. The migration, at the higher Is ratio (75%), sets the segmental mobility similar to that of a neat PIS. Even more decelerated (and constrained) is the segmental dynamics of neat PISSu. Employing known routes, from the data in Figure 11a, we could evaluate the dielectric glass transition temperature, *T*_g,diel_, and the fragility index of *α* relaxation, mα [68,79]. *T*_g,diel_ is actually the *T* extrapolation of the time scale of *α* to the equivalent frequency of conventional DSC (i.e., ~0.0016 Hz), arising from the corresponding relaxation time at *T* = *T*_g_ (*τ*_rel_~100 s).

*T*_g,diel_ and *m*_α_ were plotted in Figure 11b as a function of the Is molar fraction. Similarly to the calorimetric *T*_g_, the *T*_g,diel_ increases almost linearly with the Is fraction, from −16 to 56 °C. The calorimetric and dielectric data are in quite good agreement with each other. Taking a careful look at the main Figure 11b, there seems to be an interesting variation of the *T*_g_(Is) above the Fox prediction for the higher EG/IS ratios. This may suggest the involvement of more interactions between the two polymers that lead to constraints on the chains’ mobility or/and the chain-chain associations. The fragility index in the inset to Figure 11b shows an in general increasing *m*_α_(Is) trend (from 127 to 160), nevertheless not strictly monotonic. The increasing of *T*_g_(Is) in terms of the increasing of the dynamical constraints seems initially controversial to the ma(Is). The dynamical fragility of ‘simple’ homopolymers could be used as a measure of the chains’ cooperativity [79]. In our case, this seems to increase, despite the increasing constraints imposed by the addition of the ‘hard polymer (PISSu)’ to the softer one (PESu). During the copolymers’ synthesis, the average *M*_n_ decreases (Table 1), which would also lead to a tendency for *m*_α_ to drop. This would be due to an increase in the average cooperativity length [80,81], or, in other words, the inter-chain distance and the free volume would increase [82]. Obviously, this is not the main case here. So, taking into account the overall findings, we may conclude that the data revealed in Figure 11 suggest the implementation of more than one parameter for the molecular mobility effects. In particular, with the addition of Is, both constraints increase (hardening of the matrix); however, there seems to be an overall densification of the polymer (decrease of the free volume).

### 3.4. Thermal Stability and Degradation Mechanism of Copolymers

**Figure 12:**
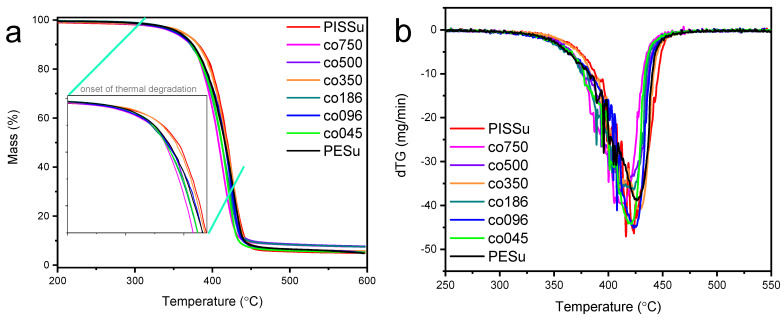
(**a**) Mass loss curves and (**b**) derivative of the mass loss curves (dTG) for neat PESu, PISSu, and the studied copolymers.

Aiming to investigate the thermal stability of the synthesized copolymers, TGA analysis was employed. The TGA thermograms of PESu, PISSu, and their copolymers are presented in Figure 12. The thermal degradation evolved in one step for all samples, in particular, starting after 350 °C and being almost completed at around 480 °C. As noted from the recorded thermograms, the studied copolymers exhibited relatively good thermal stability, with no significant mass loss up to 300 °C, implying by these means their potential to undergo shape-forming processes without degrading. Seeking further systematic behavior of thermal degradation, from the data in Figure 12 (and inset), it can be stated that there was no direct effect of the PESu/PISSu composition. The weaker thermal stability was observed for co750, whereas the higher one was observed for co096, presenting even higher thermal stability than the starting polyester building blocks. Obviously, this suggests the multiple parameters involving, beside the structure, the interchain associations (bond strengths) as well as, in connection to the previous section, the potential alterations in the free volume.

While TGA was used to study the effect of different contents in homo-polyesters on the thermal stability of the synthesized polymers, the technique of pyrolysis combined with gas chromatography/mass spectrometry (Py–GC/MS) was employed to evaluate in detail the mechanism of the thermal degradation of copolymers and, moreover, to determine whether and how the latter was altered by changing the EG/Is ratio. Analytical pyrolysis has been extensively investigated to explore the polymers’ thermal degradation mechanisms, since it delivers significant data on the macromolecules after the analysis and identification of their evolved thermal degradation products [83]. In the context of the present study, flash pyrolysis was performed at 450 °C, corresponding roughly to the end of the degradation for all studied polymers and being a temperature at which most of the degradation products were possibly formed. Understanding the decomposition process of newly synthesized polymers is crucial because it aids in selecting the most appropriate methodologies or additives to ameliorate their thermal stability.

**Figure 13:**
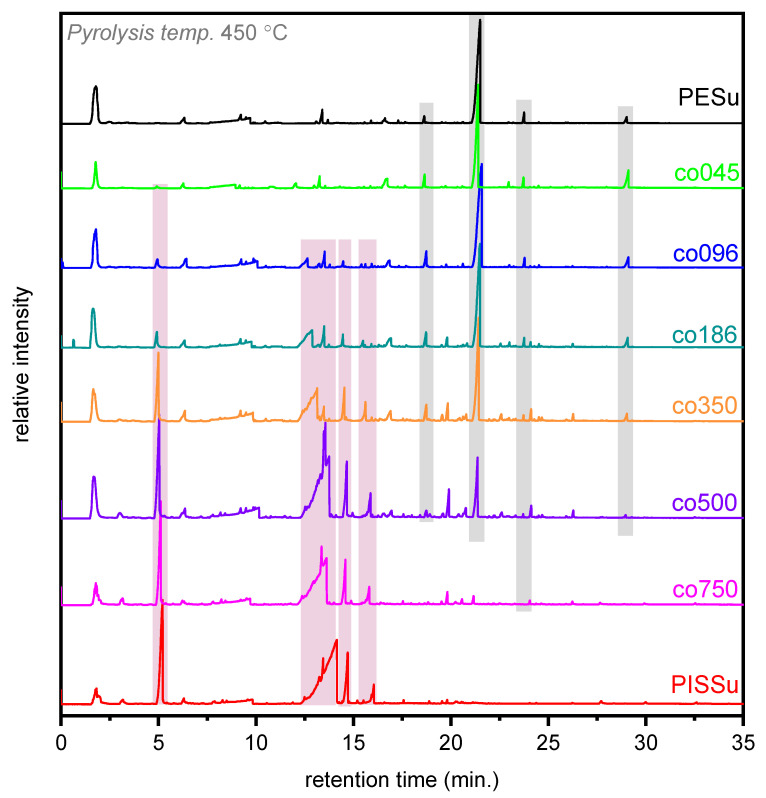
Overall ion chromatograms (TICs) of the PESu-PISSu copolymers in different ratios after pyrolysis at 450 °C.

The total ion chromatograms (TICs) of PISSu and PESu polyesters, along with their copolyesters, after pyrolysis at 450 °C are illustrated in Figure 13 (mass spectra not shown for briefness reasons). As depicted, the chromatograms of the studied samples exhibit relatively simple patterns, with some differences being present in the intensity of the peaks corresponding to each different block of polyester. Each peak corresponds to a mass spectrum that resembles a thermal decomposition product. As for the assigned compounds identified by their mass spectra and the available library, the most important pyrolysates are summed and listed in Table 3. Our research group has already thoroughly explored the thermal degradation pattern of several succinate copolymers [83,84,85], as well as the mechanism of thermal degradation of poly(isosorbide furanoate) (PIsF) [58].

At lower retention times (Rt), volatile molecules with lower molecular weights, such as carbon dioxide or acetaldehyde, were observed. The presence of carbon dioxide indicated the occurrence of homolytic processes and the decarboxylation of carboxyl-end groups in the synthesized polymers under study [86]. As the Rt proceeded, larger and more complex structures were observed. The chromatogram of PESu, as already reported in previous works, illustrated a fairly simplistic pattern, with some major pyrolytic peaks (annotated with gray color) that were identified as propanoic acid, 2-hydroxyethyl ester (Rt = 6.33 min), succinic anhydride (Rt = 9.21 min), butanedioic acid, diethyl ester (Rt = 13.40 min), allyl (2-hydroxyethyl) succinate (Rt = 16.61 min), 2-hydroxyethyl vinyl succinate (Rt = 18.62 min), 2-(acryloyloxy)ethyl (2-hydroxyethyl) succinate (Rt = 21.49 min), 2-hydroxyethyl (2-((4 oxobutanoyl)oxy)ethyl) succinate (Rt = 23.73 min), 2-((4-(2-hydroxyethoxy)-4-oxobutanoyl)oxy)ethyl vinyl succinate (Rt = 29.01 min). These compounds could be categorized as hydroxyl-, vinyl-, methyl-, and aldehyde-terminated, which can be primarily products of *β*-hydrogen and, to a lesser extent, *α*-hydrogen scission reactions. After the incorporation of Is units in the macromolecular chain of PESu, new peaks emerge in the recorded TICs. It is important to mention that the new Is-derived peaks increased in number as well as in intensity as the PISSu molar ratio in the copolymers increased, whereas the intensity of the PESu-derived peaks decreased significantly. The identified compounds assigned to PISSu counterpart are the following (annotated with pink color): tetrahydrofuran-3-ol (Rt = 1.92 min), 3a,6a-dihydrofuro[3,2-b]furan (Rt = 5.20 min), hexahydrofuro[3,2-b]furan-3-ol (Rt = 9.67 min), isosorbide (Rt = 13.42 min), 3-methoxyhexahydrofuro[3,2-b]furan (Rt = 14.70 min), and (3R,6S)-6-methoxyhexahydrofuro[3,2-b]furan-3-ol (Rt = 16.05 min). For higher retention times, pyrolysis compounds were not recorded. Noteworthy was also the formation of new peaks for almost all the studied copolymers, which were comprised of both copolymers that were absent in PESu and PISSu polyesters. These newly formed peaks were detected for Rt > 19 min and were primarily vinyl-, carboxyl-, and hydroxyl-terminated compounds, namely (((3S,6R)-6-hydroxyhexahydrofuro[3,2-b]furan-3-yl)oxy)methyl acrylate (Rt = 19.8 min), 4-((((3S,6R)-6-(hydroxymethoxy)hexahydrofuro[3,2-b]furan-3-yl)oxy)methoxy)-4-oxobutanoic acid (Rt = 24.01 min) and 2,3,3a,6a-tetrahydrofuro[3,2-b]furan-3-yl(2-((vinyloxy)methoxy)ethyl) succinate (Rt = 26.25 min). The detection of the aforementioned compounds could also be considered a validation of the successful copolymerization of the studied polymers, nicely supplementing the indirect evidence reported in the previous sections.

A large sum of interesting data regarding these novel PESu/PISSu copolyesters was recorded, as expected, with a few points still open. Further work on these materials (mechanical tests, compostability, permeation to water and gases) would be illuminating, both from the point of view of basic research and that of future applications, processing, and disposal aspects.

## 4. Conclusions

We demonstrate a sum of structural and thermodynamic data on a newly synthesized series of copolymers based on two renewable polymers, PESu and PISSu. The synthesized copolymers exhibited, in general, good thermal stability, as indicated by TGA thermograms, which is quite an exploitable parameter regarding processing and specific applications. The thermal degradation mechanism for the studied copolymers was also explored in high detail employing analytical pyrolysis. With the increasing of the Isosorbide(Is)/EG molar ratio, i.e., gradually from 5/95 to 75/25 during the in-situ copolymerization, the molar mass (copolymer chain length, *M*_n_) decreases from ~80 down to ~5 kg/mol. Despite that, the ‘single’ glass transition temperature increases monotonically from −16 to 40 °C, while, at the same time, the crystallizability of PESu vanishes. Both direct and indirect results are dependent on the homogeneity of these systems. During the molecular dynamics mapping, the local mobility was found to be dominated by the ester dipolar motions exhibiting mild effects, while the dynamical view resembles mainly that of PESu rather than that of PISSu. On the other hand, in agreement with the calorimetric findings, the dynamical glass transition was found to monotonically increase when increasing Is. Interestingly, this increase follows quite well the predictions of models on miscible polymers in the form of blends. Simultaneously, the dynamical fragility increases, supplying indirect evidence for a decrease in free volume, or, in other words, a densification of the copolymers. Combining all the results, we gained firm evidence for the ‘hardener’ role played by Is on the softer PESu, which was actually the main scope of these PESu/PISSu systems, envisaging specific applications for the future.

## Figures and Tables

**Table 1:** Code naming of the materials under investigation and properties of main interest: intrinsic viscosity, [*η*], molecular weight, *M_n_*, polydispersity index, *PDI*, average sequence length, *L*_i_, and degree of randomness, *R*, respectively (details in the main text).

					Microstructure		
Sample Code Name	Is/PESu Ratio	[*η*] (dL/g)	*M_n_* (g/mol)	*PDI*	*L_EGSu_*	*L_IsSu_*	*R*
PESu	0/100	0.51	83 k	1.71	-	-	-
co045	4.5/95.5	0.56	62 k	1.84	-	-	-
co096	9.6/90.4	0.43	39 k	2.15	-	-	-
co186	18.6/81.4	0.50	33 k	2.32	-	-	-
co350	35/65	0.18	12 k	1.83	2.7	1.7	0.97
co500	50/50	0.28	8 k	1.97	1.9	2.1	1.02
co750	75/25	0.17	7 k	1.78	1.4	3.9	0.99
PISSu	100/0	0.16	5 k	1.81	-	-	-

**Table 2:** Main thermal events as observed by DSC: glass transition temperature, *T*_g_; corresponding changes in heat capacity, Δ*c*_p_; melt crystallization temperature and enthalpy change, *T*_c_ and Δ*H*_c_, respectively; cold crystallization temperature and enthalpy change, *T*_cc_ and Δ*H*_cc_, respectively; and melting temperature and enthalpy change, *T*_m_ and Δ*H*_m_, respectively.

	DSC Cooling			DSC Heating					
Sample Code Name	*T*_c_ (°C)	Δ*H*_c_ (J/g)	*T*_g_ (°C)	*T*_g_ (°C)	Δ*c*_p_ (J/g∙K)	*T*_cc_ (°C)	Δ*H*_cc_ (J/g)	*T*_m_ (°C)	Δ*H*_m_ (J/g)
PESu	34	1	−16	−13	0.65	41/81	51	102	53
co045	-	-	−12	−9	0.63	61	12	94	12
co096	-	-	−9	−6	0.60	-	-	-	-
co186	-	-	−2	1	0.59	-	-	-	-
co350	-	-	3	5	0.56	-	-	-	-
co500	-	-	19	20	0.52	-	-	-	-
co750	-	-	40	43	0.49	-	-	-	-
PISSu	-	-	51	53	0.46	-	-	-	-

**Table 3:** Analysis of evolved gaseous compounds during pyrolysis at 450 °C of neat PESu, PISSu, and their copolymers at different content ratios.

Rt (min.)								MW (amu)	Assigned Structure
PISSu	co750	co500	co350	co186	co096	co045	PESu		
1.72	1.72	1.68	1.67	1.64	1.77	1.78	1.78	44	CO_2_ or acetaldehyde
1.92	1.91	n.d.	n.d.	n.d.	n.d.	n.d.	n.d.	86	(S)-tetrahydrofuran-3-ol 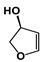 or (R)-tetrahydrofuran-3-ol 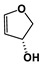
5.20	5.11	5.04	5.00	4.91	4.64	n.d.	n.d.	110	3a,6a-dihydrofuro[3,2-b]furan 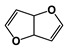
n.d.	n.d.	6.32	6.35	6.34	6.42	6.28	6.33	118	propanoic acid, 2-hydroxyethyl ester 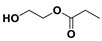
n.d.	n.d.	9.20	9.20	9.22	9.24	9.25	9.21	100	succinic anhydride 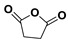
9.67	9.69	9.69	9.50	9.49	n.d.	n.d.	n.d.	126	(3R)-hexahydrofuro[3,2-b]furan-3-ol 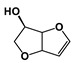 or (3S)-hexahydrofuro[3,2-b]furan-3-ol 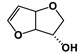
n.d.	n.d.	n.d.	13.47	13.49	13.51	13.25	13.40	172	butanedioic acid, diethyl ester 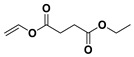
13.42	13.36	13.32	13.49	13.51	13.50	13.55	n.d.	146	hexahydrofuro[3,2-b]furan-3,6-diol (isosorbide) 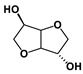
14.70	14.60	14.64	14.53	14.45	14.49	n.d.	n.d.	142	(3S)-3-methoxyhexahydrofuro[3,2-b]furan 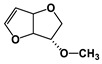
16.05	15.80	15.87	15.61	15.49	n.d.	n.d.	n.d.	160	(3R,6S)-6-methoxyhexahydrofuro[3,2-b]furan-3-ol 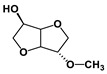
n.d.	n.d.	16.94	16.89	16.91	16.83	16.72	16.61	202	allyl (2-hydroxyethyl) succinate 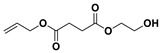
n.d.	n.d.	18.71	18.71	18.71	18.72	18.63	18.62	188	2-hydroxyethyl vinyl succinate 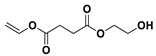
n.d.	19.81	19.88	19.83	19.80	19.75	n.d.	n.d.	229	(((3S,6R)-6-hydroxyhexahydrofuro[3,2-b]furan-3-yl)oxy)methyl acrylate 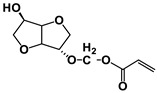
n.d.	21.15	21.36	21.42	21.47	21.56	21.40	21.49	260	2-(acryloyloxy)ethyl (2-hydroxyethyl) succinate 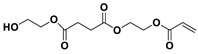
n.d.	n.d.	23.71	23.72	23.74	23.76	23.70	23.73	290	2-hydroxyethyl (2-((4 oxobutanoyl)oxy)ethyl) succinate 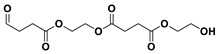
n.d.	24.03	24.10	24.10	24.08	n.d.	n.d.	n.d.	306	4-((((3S,6R)-6-(hydroxymethoxy)hexahydrofuro[3,2-b]furan-3-yl)oxy)methoxy)-4-oxobutanoic acid 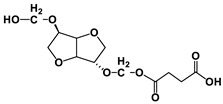
n.d.	26.21	26.25	26.24	n.d.	n.d.	n.d.	n.d.	328	2,3,3a,6a-tetrahydrofuro[3,2-b]furan-3-yl (2-((vinyloxy)methoxy)ethyl) succinate 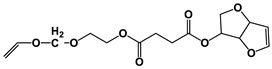
n.d.	n.d.	28.95	29.02	29.05	29.10	29.10	29.01	332	2-((4-(2-hydroxyethoxy)-4-oxobutanoyl)oxy)ethyl vinyl succinate 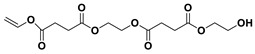

## Data Availability

The original contributions presented in the study are included in the article, further inquiries can be directed to the corresponding authors.
